# Treatment of withdrawal headache in patients with medication overuse headache: a pilot study

**DOI:** 10.1186/s10194-017-0763-9

**Published:** 2017-05-12

**Authors:** Sabina Cevoli, Giulia Giannini, Valentina Favoni, Rossana Terlizzi, Elisa Sancisi, Marianna Nicodemo, Stefano Zanigni, Maria Letizia Bacchi Reggiani, Giulia Pierangeli, Pietro Cortelli

**Affiliations:** 1 IRCCS Institute of Neurological Sciences of Bologna, UOC Clinica Neurologica, Bellaria Hospital, Via Altura 3, 40139 Bologna, Italy; 2 Department of Biomedical and NeuroMotor Sciences (DiBiNeM), Alma Mater Studiorum - University of Bologna Italy, Bologna, Italy; 3 Neurology, AUSL (Local Health Service) of Ferrara, Ferrara, Italy; 4 Division of Neurology, Maggiore Hospital, IRCCS Institute of Neurological Sciences of Bologna, Bologna, Italy; 5 Functional MR Unit, Policlinico S.Orsola-Malpighi, Bologna, Italy; 6 Department of Experimental, Diagnostic and Specialty Medicine (DIMES), Alma Mater Studiorum, University of Bologna, Bologna, Italy

**Keywords:** Medication overuse headache, Detoxification, Migraine

## Abstract

**Background:**

Drug withdrawal still remains the key element in the treatment of Medication Overuse Headache (MOH), but there is no consensus about the withdrawal procedure. Still debated is the role of the steroid therapy. The aim of this study was to evaluate the effectiveness of methylprednisolone or paracetamol in the treatment of withdrawal headache in MOH.

**Methods:**

We performed a pilot, randomized, single-blinded, placebo controlled trial. MOH patients, unresponsive to a 3 months prophylaxis, underwent withdrawal therapy on an inpatient basis. Overused medications were abruptly stopped and methylprednisolone 500 mg i.v (A) or paracetamol 4 g i.v. (B) or placebo i.v. (C) were given daily for 5 days. Patients were monitored at 1 and 3 months.

**Results:**

Eighty three consecutive MOH patients were enrolled. Fifty seven patients completed the study protocol. Nineteen patients were randomized to each group. Withdrawal headache on the 5th day was absent in 21.0% of group A, in 31.6% of group B and in 12.5% of group C without significant differences. Withdrawal headache intensity decreased significantly after withdrawal without differences among the groups. Rregardless of withdrawal treatment, 52% MOH patients reverted to an episodic migraine and 62% had no more medication overuse after 3 months.

**Conclusions:**

This study suggests that in a population of severe MOH patients, withdrawal headache decreased significantly in the first 5 days of withdrawal regardless of the treatment used. Methylprednisolone and paracetamol are not superior to placebo at the end of the detoxification program.

## Background

Medication Overuse Headache (MOH) is a worsening of a pre-existing primary headache associated with overuse of acute headache medication [1]. MOH has a strong social impact and represents a public-health concern given the large amount of associated disability and financial costs [2]. MOH affects between 1 and 2% of the general population [3] and 30–50% of patients seen in headache centres. In tertiary headache centres we are used to visit refractory patients with MOH and abrupt drug withdrawal is actually considered the best treatment [4]. Although stopping the acute medication may result in withdrawal symptoms such as increase of headache, nausea, vomiting, arterial hypotension, tachycardia, insomnia and anxiety [5], subsequent headache improvement usually, but not always, occurs. According to EFNS guidelines, treatment of MOH patients should include: patient’s education on the nature of the disease, on risk factors and on treatment options; withdrawal including rescue medication; preventive treatment and a multimodal approach including psychological support, if necessary [6].

However, the role of detoxification programs and the possibility to use only the prophylactic therapy is still highly debated [7, 8]. Previous studies have shown that simple information about MOH may be sufficient for some treatment-naïve patients to stop medication overuse on their own [9–11].

MOH subjects who fail withdrawal after simple advice or are complicated by a long duration of disease, multiple overuse, comorbidity or history of unsuccessful treatments have been scarcely studied [4]. However, a study showed that 49% of patients who failed to withdraw from medication overuse after simple advice, had a successful outcome after a structured detoxification program and close follow-up [12].

Another unsolved issue regards whether to begin prophylactic treatment immediately or after the effect of the detoxification, although, as recently revised, the combination of education and prophylactic treatment is superior to prophylactic treatment alone [4].

A multinational study on a large population applied a consensus protocol for the management of MOH showing that two-thirds of subjects were no longer overusers after 6 months and in 46.5% headache reverted to an episodic pattern. Dropout rate was higher in the outpatient program when compared with the inpatient approach, but both regimens were effective [13]. Moreover disability, depression and anxiety were considerably reduced in patients with MOH after a protocol based on rescue, symptomatic and prophylactic medications [14].

Treatment recommendations for the acute phase of drug withdrawal vary considerably among studies. They include fluid replacement, analgesics, anxiolytics, neuroleptics, amitriptyline, valproate, intravenous dihydroergotamine, oxygen and antiemetics. Still debated is the role of steroid therapy [15]. Two independent placebo-controlled randomized studies revealed discordant results regarding the efficacy of the oral prednisone therapy in controlling withdrawal symptoms and headache intensity in the first six and 5 days of withdrawal respectively [16, 17]. More recently, a study partly supported the hypothesis that prednisone reduces the consumption of rescue medications without decreasing the severity and duration of withdrawal headache [18], but comparisons with safer and better tolerated analgesics are lacking.

We aimed to perform a pilot study in order to evaluate the efficacy of methylprednisolone or paracetamol on withdrawal headache in MOH patients.

## Methods

### Standard protocol approvals and patient consents

The study was conducted in agreement with principles of good clinical practice and the study protocol was approved by the Local Ethic Committee of the local health service of Bologna, Italy (n. 504/CE). All patients gave their written informed consent to study participation.

### Participants

Patients from the Headache Centre of IRCCS of Neurological Sciences of Bologna, Italy were recruited consecutively. Patients were eligible if they were ≥18 years of age, were able to give verbal and written informed consent and met criteria for MOH as defined by the International Headache Society in 2006 [19]: headache present on ≥15 days/months, regular overuse for >3 months of ergotamine, triptans, opioids or combination analgesics on ≥10 days/months, or simple analgesics or any combination of ergotamine, triptans, combination analgesics or opioids on ≥10 days/months. Exclusion criteria included pregnancy and breast-feeding, secondary headaches, history of other types of addiction (such as alcohol, sedative, cannabis and psychoactive substances), as well as any serious ongoing physical or psychiatric illness. Patients with contraindication to use steroids or paracetamol, overusing paracetamol or using steroids for comorbidities were also excluded. Secondary headaches were ruled out by clinical examination, laboratory testing and neuroimaging studies, when indicated.

### Study design and procedure

**Fig. 1:**
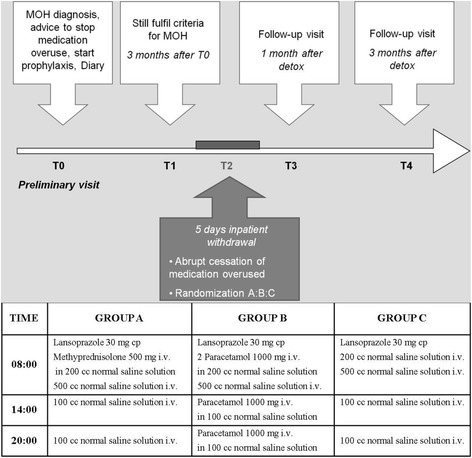
Study design and treatment groups. A: Methyprednisolone group; B: Paracetamol group; C: Placebo group; *MOH* Medication Overuse Headache

The study was a pilot, randomized, single-blind, placebo controlled trial. Fig. 1 illustrates the study design. MOH patients, unresponsive to education on the nature of the disease, simple advice to reduce the intake of medication and a prophylaxis in a three-month run-in period, underwent withdrawal therapy on an inpatient basis of 5 days. Overused medications were suddenly stopped and patients were randomized (1:1:1) to methylprednisolone (group A) or paracetamol (acetaminophen) (group B) or placebo (group C) for 5 days. Patients in group A received methylprednisolone 500 mg i.v. once a day, patients in group B received paracetamol 2000 mg at 8.00 a.m., 1000 mg at 2.00 p.m., and 1000 mg at 8.00 p.m., and patients in group C received normal saline solution i.v. All patients received lansoprazole 30 mg cap per os at 8.00 a.m. Treatment groups were scheduled in order to maintain patients blind through a double dummy design (Fig. 1). Allowed rescue therapies were: metoclopramide 10 mg i.m. and lorazepam 1 mg or 2.5 mg cap.

Subjects were assigned sequentially to group A or B or C when admitted to hospital receiving a computer-generated random medication code number. The random allocation sequence was not generated by researchers who assigned participants to interventions. Subjects were kept blind about the assigned treatment until discharge.

### Study visits

Visits occurred at baseline (preliminary visit T0), 3 months after baseline (T1), 5 days after inpatient withdrawal program (T2), then at 1 month (T3) and 3 months (T4) after inpatient withdrawal program (Fig. 1). At T0 patients were educated about the MOH diagnosis and received advice to stop overused drugs; a pharmacological prophylaxis was prescribed.

Education consisted in a brief explanation about the nature of the disease and about the consequences of too frequent intake of medication to treat headache attacks. Prophylactic treatment was chosen based on the efficacy and side effects of previous treatments, comorbidity and patients’ preferences. At T1, patients still fulfilling the diagnosis of MOH were planned for the inpatient 5-day withdrawal program (T2). T3 and T4 were follow-up visits.

A clinical diary in which patients recorded all headache attacks and the drugs taken for headache during all the study period was given at the preliminary visit and checked at every follow-up visit. Patients recorded the number of days of headache attacks with daytime duration of headache (number of hours), and headache intensity (classified as 1 = mild, 2 = moderate, 3 = severe). Another daily diary was used during the inpatient period to collect data about withdrawal headache and other withdrawal symptoms together with rescue medication intake. Depressive and anxious symptoms (Zung Self-Rating Anxiety Scale and Zung Self-Rating Depression Scale) [20, 21], and degree of disability (Migraine Disability Assessment Score, MIDAS) [22] were evaluated at T0. Patients were interviewed and examined by neurologists expert in headaches.

### Outcome measures

The primary endpoint was to evaluate the efficacy of steroids or paracetamol i.v. in the treatment of withdrawal headache in patients with MOH (absence of headache at the fifth day of withdrawal).

Secondary endpoints were: headache intensity and associated withdrawal symptoms each day of treatment, number of rescue medications needed during hospitalization, efficacy of detoxification on headache frequency and medication overuse at follow-up (1 and 3 months after inpatient withdrawal). Associated withdrawal symptoms analysed included nausea, vomiting, arterial hypotension/hypertension, tachycardia, dizziness, photo-phonophobia and anxious symptoms.

### Statistics

The normality of the distribution of the parameters was checked using a Skewness-Kurtosis test. Quantitative variables were expressed as the mean ± standard deviation (SD) or median along with interquartile ranges (IQR) when appropriate, while categorical variables were described by their absolute and/or relative frequencies. We compared categorical variables using Chi square test. Oneway Analysis of variance (ANOVA) and Kruskal-Wallis Tests were performed to compare continuous variables with a symmetrical (normal) and an asymmetrical (non-normal) distribution respectively. Post hoc test was performed when appropriate. We performed repeated measures ANOVA to compare headache intensity per day among groups (within-subjects variable time: headache intensity on days 1–2–3-4-5; among subjects variable group: A vs. B vs. C; and interaction between days and treatment groups). Significance level was set at *p* ≤ 0.05. Data analysis was performed with STATA® version 12.0.

This is a pilot study because any previous study with the same primary endpoint was published at the time of the beginning of our study, so a power analysis was not performed.

## Results

**Fig. 2:**
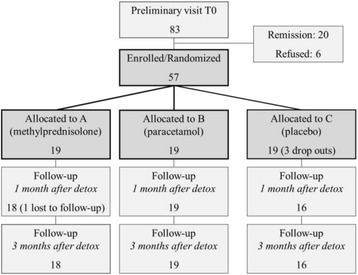
Flow chart of patients included in the study

At baseline, 83 consecutive patients were enrolled for the study; 26 were excluded because they did not meet the inclusion criteria: 20 recovered with prophylactic therapy and the education during the run-in period, while 6 refused hospitalization (Fig. 2). Of the 57 enrolled patients, 50 (87.7%) were females and 7 (12.3%) were males; mean age ± standard deviation (SD) was 47.3 ± 10.3 years. All participants suffered from migraine at onset, with a mean age at onset ± SD was 15.0 ± 7.1 years, and a chronification age of 36.0 ± 9.5 years (Table 1).

**Table 1:** Demographic and baseline clinical characteristics of the study sample

		Total	Withdrawal therapy groups			
			A: Methylprednisolone	B:Paracetamol	C:Placebo	*p* value
Sample	N (%)	57	19 (33.3)	19 (33.3)	19 (33.3)	
Age (years)	mean ± SD	47.3 ± 10.3	45.7 ± 9.5	49.8 ± 10.4	46.5 ± 11.2	0.4402
Sex						
Males	N (%)	7 (12.3)	2 (10.5)	2 (10.5)	3 (15.8)	0.850
Females	N (%)	50 (87.7)	17 (89.5)	17 (89.5)	16 (84.2)	
Marital Status						
Single	N (%)	7 (12.3)	3 (15.8)	3 (15.8)	1 (5.3)	0.842
Married	N (%)	43 (75.4)	15 (78.9)	13 (68.4)	15 (78.9)	
Separated/Divorced	N (%)	5 (8.8)	1 (5.3)	2 (10.5)	2 (10.5)	
Widower	N (%)	2 (3.5)	0 (0.0)	1 (5.3)	1 (5.3)	
Years of Education	mean ± SD	12.5 ± 4.2	12.1 ± 3.5	12.7 ± 5.4	12.8 ± 3.6	0.8575
Employment						
Unemployed	N (%)	1 (1.7)	0 (0.0)	1 (5.3)	0 (0.0)	0.859
Student	N (%)	3 (5.3)	1 (5.3)	1 (5.3)	1 (5.3)	
Employee	N (%)	34 (59.7)	13 (68.4)	9 (47.4)	12 (63.2)	
Housewife	N (%)	11 (19.3)	4 (21.0)	4 (21.0)	3 (15.8)	
Retired	N (%)	4 (7.0)	0 (0.0)	2 (10.5)	2 (10.5)	
Self-employed	N (%)	4 (7.0)	1 (5.3)	2 (10.5)	1 (5.3)	
Age at Migraine Onset (years)	mean ± SD	15.0 ± 7.1	13.8 ± 4.5	15.7 ± 8.5	15.3 ± 7.8	0.7029
Age of Migraine chronification	mean ± SD	36.0 ± 9.5	34.3 ± 8.7	39.8 ± 10.2	33.8 ± 8.9	0.0953
Duration of MOH (years)	med; IQR	10; 3–14	11; 3–15	8; 3–12	10; 3–14	0.8198
Headache frequency (days/month)	med; IQR	28.5; 21–30	29; 21–30	24.5; 20–30	30; 21–30	0.4243
Headache duration (hours/day)	mean ± SD	8.6 ± 5.8	7.0 ± 5.2	6.8 ± 4.3	12.1 ± 6.3	**0.0230**
Headache intensity (1–3 scale)	mean ± SD	1.6 ± 0.5	1.7 ± 0.5	1.7 ± 0.5	1.5 ± 0.4	0.3555
Frequency of medication	mean ± SD	23.4 ± 6.7	23.4 ± 7.2	23.4 ± 5.8	23.2 ± 7.2	0.9941
intake (days/month)						
Overused Drugs						
Triptans	N (%)	39 (68.4)	13 (68.4)	13 (68.4)	13 (68.4)	1
Simple analgesics and/or NSAIDs	N (%)	18 (31.6)	4 (21.0)	8 (42.1)	6 (31.6)	0.377
Ergots	N (%)	3 (5.3)	2 (10.5)	1 (5.3)	0 (0.0)	0.348
Combination analgesics	N (%)	17 (29.8)	7 (36.8)	6 (31.6)	4 (21.0)	0.556
Previous detoxification						
No	N (%)	32 (56.1)	10 (52.6)	12 (63.2)	10 (52.6)	0.180
Yes, outpatient program	N (%)	3 (5.3)	1 (5.3)	1 (5.3)	1 (5.3)	
Yes, inpatient program	N (%)	19 (33.3)	8 (42.1)	3 (15.8)	8 (42.1)	
Yes, inpatient and outpatient programs	N (%)	3 (5.3)	0 (0.0)	3 (15.8)	0 (0.0)	
Migraine disability assessment score	med; IQR	80; 35–130	59.5; 21.5–156.5	83.5; 36.5–170	69; 41–91	0.7270
Epworth Sleepiness Scale	mean ± SD	6.3 ± 3.5	7.2 ± 3.6	5.6 ± 4.1	6.2 ± 2.8	0.4303
Zung Self-Rating Anxiety Scale	med; IQR	35; 33–39	35; 32.5–42.5	33.5; 32–35	37; 34–38	0.2035
Zung Self-Rating Depression Scale	mean ± SD	44.6 ± 8.9	46.7 ± 10.1	42.9 ± 7.5	43.8 ± 8.9	0.4082

*Legend*: *IQR* interquartile range; med: median; *MOH* medication overuse headache; *N* sample size; *NSAIDs* Nonsteroidal Anti-inflammatory Drugs; *SD* standard deviation

Statistically significant *p*-values are denoted in bold

Overused medications included triptans (68.4%), simple analgesics (31.6%), ergots (5.3%) and combination analgesics (29.8%). No one overused opiates. Of the final sample, 24 (42.1%) patients received preventive monotherapy while 33 (57.9%) received polytherapy: 21 (36.8%) received a combination of 2 drugs and 12 (21.1%) of 3 drugs. Main prophylactic medications included amitriptyline (26.3%), beta-blockers (29.8% atenolol and 8.8% propranolol), flunarizine (7.0%), perphenazine (12.3%), topiramate (28.1%), valproic acid (8.8%). No differences in prophylactic medications were found among the three groups.

Table 1 presents the demographic and clinical characteristics of the patients randomized to the three different detoxification groups: 19 patients were randomized to A, 19 to B and 19 to C. Sociodemographic variables, headache frequency (days per month), headache intensity, frequency of overused medications (days per month), MOH duration (years), previous detoxification and scores at Zung scales and MIDAS did not differ significantly among the three groups. Participants randomized to group C showed an increased headache duration (hours/day) when compared to those randomized to others groups (*p* = 0.0230): an ANOVA post hoc test showed that this statistical significance was attributable to the difference between B vs. C group (*p* = 0.042).

Three patients (2 females and 1 male) randomized to C group dropped out during the 3rd day of hospitalization. Withdrawal headache on the 5th day was absent in 4 patients (21.0%) of group A, in 6 patients (31.6%) of group B and 2 (12.5%) of group C without significant differences (*p* = 0.396) (Table 2).

**Table 2:** Clinical features of patients randomized to the three different detoxification groups during the withdrawal program

		Withdrawal therapy groups			
		A:Methylprednisolone	B:Paracetamol	C:Placebo	*p* value
Headache Intensity (1–3 scale)					
1st day	mean ± SD	1.8 ± 0.7	1.5 ± 0.9	2.0 ± 0.7	
2nd day	mean ± SD	1.8 ± 0.5	1.9 ± 0.8	2.3 ± 0.5	< **0.001** ^a^
3rd day	mean ± SD	1.3 ± 0.7	1.5 ± 0.8	1.9 ± 0.9	0.103^b^
4th day	mean ± SD	1.4 ± 0.6	1.4 ± 0.8	1.2 ± 0.7	0.192^c^
5th day	mean ± SD	1.2 ± 0.8	1.1 ± 0.9	1.1 ± 0.6	
Headache on 5th day (yes/no)	N (%) / N (%)	15 (79.0) /4 (21.0)	13 (68.4) / 6 (31.6)	14 (87.5) / 2 (12.5)	0.396
Associated withdrawal Symptoms					
1st day	N (%)	15 (79.0)	13 (68.4)	15 (79.0)	0.685
2nd day	N (%)	18 (94.7)	14 (73.7)	15 (79.0)	0.207
3rd day	N (%)	13 (68.4)	15 (79.0)	10 (62.5)	0.554
4th day	N (%)	15 (79.0)	14 (73.8)	8 (50.0)	0.154
5th day	N (%)	12 (63.2)	10 (52.6)	6 (37.5)	0.317
Number of Medication Intake	med; IQR	3; 2–6	2; 0–3	4; 1–6	0.139
Withdrawal headache duration (days)	med; IQR	7; 5–7	6; 5–8	7; 6.5–8	0.5797

*Legend*: *IQR* interquartile range; med: median; *N* sample size; *SD* standard deviation

^a^from testing headache intensity for all patients across days

^b^from testing headache intensity among treatments

^c^from testing the interaction between treatments and days of headache intensity

Statistically significant *p*-values are denoted in bold

Withdrawal headache intensity decreased significantly after withdrawal without differences among the three groups (headache intensity, days-effect: *p* < 0.001, F = 13.25; group effect: *p* = 0.103, F = 2.30; interaction days-group effects: *p* = 0.192, F = 1.41) (Table 2). The highest rebound headache intensity was reached during the 2nd day of withdrawal. Headache intensity was lower in A and B vs. C in the 2nd day, as showed in Table 2, without reaching a significantly difference.

According to the intention-to-treat analysis, withdrawal headache intensity decreased significantly after withdrawal with significant differences among the three groups (headache intensity, days-effect: *p* < 0.001, F = 10.00; group effect: *p* = 0.002, F = 6.17) without differences when considering their interaction (headache intensity, interaction days-group effects: *p* = 0.508, F = 0.91).

The three groups did not differ in associated withdrawal symptoms and in number of rescue medications according to the per-protocol analysis (Table 2). Any serious adverse events have not been reported.

Excluding patients chronic at T3, the median (IQR) of the withdrawal headache duration was 7 days (5–8) without differences among treatment groups (Table 2). After the hospitalization one patient randomized to group C was lost at 1 month follow-up. Of the 53 remaining patients, 33 (62.2%) returned to suffer with less than 15 migraine days in the first month after detoxification. And 39 (73.6%) stopped to overuse medications, with no detectable differences among groups. Overall headache frequency was reduced to a median (IQR) of 13.5 (8–24) while frequency of medication intake was reduced to a median (IQR) of 8 (5–13) without differences among groups (Table 3). After the 3 months of follow-up, 28 (52.8%) participants still presented an episodic migraine: 9 (50.0%) randomized to group A, 8 (42.1%) to B and 11 (68.7%) to C without significant differences. Of the final sample, 33 (62.3%) subjects were MOH-free without differences among groups: 11 (61.1%) randomized to A, 9 (47.4%) to B and 13 (81.2%) to C group (Table 3).

**Table 3:** Clinical features of patients randomized to the three different detoxification groups at follow-up visits

		Total	Withdrawal therapy groups			
			A:Methylprednisolone	B:Paracetamol	C:Placebo	*p* value
Sample	N (%)	53	18	19	16	
Headache frequency						
< 15 days T4	N (%)	28 (52.8)	9 (50.0)	8 (42.1)	11 (68.7)	0.481
≥ 15 days at T3	N (%)	20 (37.8)	8 (44.4)	8 (42.1)	4 (25.0)	
≥ 15 days at T4	N (%)	5 (9.4)	1 (5.6)	3 (15.8)	1 (6.3)	
Medication overused after detoxification						
< 15 days T3	N (%)	33 (62.3)	11 (61.1)	9 (47.4)	13 (81.2)	0.216
≥ 15 days at T3	N (%)	14 (26.4)	6 (33.3)	6 (31.6)	2 (12.5)	
≥ 15 days at T4	N (%)	6 (11.3)	1 (5.6)	4 (21.0)	1 (6.3)	
Headache frequency T3 (days/month)	med; IQR	13.5; 8–24	14.5; 7–26	17; 9.5–24	10; 7.5–17	0.428
Headache frequency T4 (days/month)	med; IQR	13.5; 7–20	14; 4–26	17; 7–20	12; 7–18	0.735
Frequency of Medication Intake T3 (days/month)	med; IQR	8; 5–13	8; 4–14	8.5; 6–17	7.5; 4–9.5	0.438
Frequency of Medication Intake T4 (days/month)	med; IQR	9.5; 4.5–13	10.5; 4–15	10; 5–14	9; 7–10	0.851

*Legend*: *IQR* interquartile range; med: median; *N* sample size

## Discussion

This study suggests that in a population of severe MOH patients, withdrawal headache decreased significantly in the first 5 days of withdrawal regardless of the treatment used to relieve withdrawal symptoms. No difference was found regarding associated withdrawal symptoms and in the number of rescue medications according to the per-protocol analysis, even though the number of rescue medications was lower in the two treatment groups versus placebo according to the intention-to-treat analysis. The worst headache was registered between 24 and 72 h of withdrawal program and only in the second day methylprednisolone or paracetamol indifferently appeared slightly superior to placebo. Rescue therapies were requested only in the first 3 days of withdrawal program when the headache intensity was higher. Moreover, the mean duration of rebound headache was 7 days without difference between placebo and active groups.

In the intention-to-treat analysis, withdrawal headache intensity decreased significantly after withdrawal with significant differences among the three groups (headache intensity, days-effect: *p* < 0.001, F = 10.00; group effect: *p* = 0.002, F = 6.17). This statistical significance among groups is attributable to the difference in headache intensity between treatments and placebo groups. The mean headache intensity ± SD during withdrawal was 1.51 ± 0.69 in A, 1.48 ± 0.85 in B and 1.82 ± 0.83 in C, with greater difference during the second and third days of withdrawal (second day: 2.32 ± 0.50 in A, 1.93 ± 0.75 in B and 1.84 ± 0.52 in C; third day: 2.01 ± 0.87 in A, 1.53 ± 0.79 in B and 1.28 ± 0.68 in C). According to intention-to-treat analysis we considered the headache intensity of the three patients in the placebo group that dropped out exactly in the worst day and this probably explains the differences among groups. The main responsible for the three dropouts in the placebo group is probably the lack of blindness of the neurologists. However, this significance did not remain when considering the interaction between time of withdrawal and groups (headache intensity, interaction days-group effects: *p* = 0.508, F = 0.91).

Noteworthy, regardless of withdrawal treatment, more than 60% MOH patients resistant to prophylaxis reverted to an episodic migraine and 73% had no more medication overuse after 1 month. After the 3 months of follow-up, 52% of subjects still presented an episodic migraine and 62% were no longer overusers. In addition, we found that 26% of MOH patients attending a tertiary academic headache centre recovered with simple education about the negative impact of medication overuse and prophylactic therapy prescribed during the preliminary outpatient visit. Education on MOH and drug withdrawal still remain the key elements in the treatment of MOH, but there is no consensus about the withdrawal procedure [4]. Some headache specialists prefer inpatient programs, others an outpatient setting, nevertheless previous studies revealed in both a significant decrease in headache days per month and in the score of migraine disability, ruling out the superiority of one of these two methods [23, 24]. However, inpatient withdrawal resulted significantly more effective compared to both advice alone and outpatient strategy in complicated MOH patients [9].

Very few randomized controlled studies were performed in order to verify the efficacy of pharmacological treatment on withdrawal headache. Often, patients are given a short course of steroids at different dosages and route of administration. In 2008 Pageler and co-authors in a small randomized, placebo controlled, double blind study, reported that prednisone 100 mg given orally once a day for the first 5 days of inpatient withdrawal treatment reduced significantly the total number of hours with severe or moderate headache within the first 72 and 120 h [17]. In the same year Bøe and colleagues performed a randomized, double blind, placebo controlled study in order to verify whether oral prednisolone reduced headache intensity during the first 6 days after medication withdrawal. The patients were hospitalized for the first 3 days and were randomized to prednisolone 60 mg on days 1 and 2, 40 mg on days 3 and 4 and 20 mg on days 5 and 6 or placebo. One hundred MOH patients were included, 65 of whom had migraine, 13 had tension type headache and 22 had both migraine and tension type headache. Prednisolone was not effective on rebound headache in this unselected patient group [16]. More recently, Rabe and colleagues evaluated the efficacy of 100 mg of prednisone over 5 days in the treatment of withdrawal headache. This was a multicentre double blind, placebo controlled, randomized study involving 96 MOH patients with migraine or episodic tension type headache as primary headache. Prednisone reduced rescue medication intake without decreasing the number of hours with moderate or severe headache and duration of withdrawal headache [18]. Finally, Taghdiri and co-workers compared the efficacy of 400 mg/day celecoxib for the first 5 days then decreased at a rate of 100 mg every 5 days vs prednisone 75 mg/day for the first 5 days then tapered off every 5 days in 97 MOH patients. Patients treated with celecoxib had slightly lower headache intensity at the Visual Analogue Scale during the first 3 weeks after withdrawal. However, headache frequency and the demand of rescue medications, which were the primary endpoints, did not differ among groups [25].

Our study confirms that withdrawal of medication overuse is healing regardless of the treatments of rebound headache and symptoms, but it is necessary only when education and prophylaxis fail.

In this study, prophylaxis was started simultaneously with the simple advice to stop medication overuse, so we do not know the relative weight of the two procedures. Moreover, whether to begin prophylactic treatment before, immediately or after the effect of the detoxification is an important unsolved issue.

Our suggested treatment strategy is to counsel patients with MOH and start prophylaxis that may be effective in patients with chronic migraine and medication overuse as evidenced in randomised controlled trials [26]. Moreover, recent articles reported that OnabotulinumtoxinA is effective in MOH prophylaxis, also for patients who had failed previous detoxification, and shows good tolerability and few side effects, so this treatment should be taken into consideration [27, 28]. Many patients will be able to reduce their intake of medications with reduction of headache days without other more expensive and heavy treatments. However, a structured detoxification program should be offered in a short time when the first strategy fails without wasting other time.

Several limitations of our study should be discussed. First of all, this was a single blind study because a double blind design was not feasible in our neurological ward. The lack of blindness of the neurologists was probably the main responsible for the three dropouts in the placebo group exactly in the worst day, without waiting for a possible natural improvement. Moreover, the fact to be in a tertiary centre, probably contributed to pick out more severe MOH patients with previous therapeutic failures, as evidenced in the description of baseline features. In our sample, none patient was treated with OnabotulinumtoxinA before detoxification because the enrolment in this study was close to the end when our local health service approved its use. At this time, the enrolment in the study was close to the end. Placebo in this study was a rehydration treatment that appeared to be not less effective than high doses of active i.v. drugs. Zung and MIDAS scales were lost at follow-up, so they were useful only to describe baseline features of the sample. For the same reason a stratification of patients in order to analyse possible predictors of the outcome was not performed. Finally, the sample size is relatively small but for feasibility reasons we did not recruited further patients. Therefore, we cannot exclude that the absence of statistical significance between groups may be related to the small number of patients in each group. The advantage of the study was in fact, the high homogeneity of the included patients: all were complicated and all had migraine as primary headache. MOH was diagnosed according the International Headache Society 2006 criteria [19], but a chart revision confirmed that all patients included responded to chronic migraine with MOH according ICHD-3 beta criteria [1].

In conclusion, methylprednisolone 500 mg i.v. and paracetamol (acetaminophen) 4 g/die i.v. are not superior to placebo at the end of the detoxification program. Methylprednisolone and paracetamol, a well-tolerated simple analgesic, have the same efficacy in controlling withdrawal headache but might be superior to placebo (fluid replacement) in reducing the intensity of rebound headache only during the second day of withdrawal.

About 50% of patients, resistant to prophylaxis, are no longer overusers after detoxification.

In spite of being a pilot study, present data remain however important for the implementation of further studies on this topic. However, further comparative, multicentre studies among prophylaxis and detoxification programs in MOH patients are necessary to evaluate outcomes and costs [29] in order to optimize the healthcare management of people with chronic disabling headache.

## Conclusions

Education on medication overuse and drug withdrawal still remain the key elements in the treatment of medication overuse headache. Our study suggest that Methylprednisolone and Paracetamol may be useful in reducing the intensity of rebound headache during the second day of withdrawal, but they are not superior to placebo at the end of the detoxification program.

## Abbreviations

MIDAS: Migraine Disability Assessment Score
MOH: Medication Overuse Headache
